# Global land-use intensity and anthropogenic emissions exhibit symbiotic and explosive behavior

**DOI:** 10.1016/j.isci.2022.104741

**Published:** 2022-07-14

**Authors:** Samuel Asumadu Sarkodie, Phebe Asantewaa Owusu

**Affiliations:** 1 Nord University Business School (HHN), Post Box 1490, 8049 Bodø, Norway

**Keywords:** Environmental science, Environmental health, Environmental policy, Energy policy, Energy sustainability

## Abstract

The intensification of land use is accelerating and remains a threat to achieving environmental sustainability. Although prior literature identifies unsustainable demand for resources as crucial to ecosystem vitality, we highlight explosive behavior and indicators associated with changing global land-use intensity and emissions. We assess emission footprints, forestry, and agricultural land-use intensity across income groups. We find that long-term income growth above US$1005/capita has mitigation effects on emissions, whereas emissions stimulate the global expansion of land use for agricultural and forestry activities. Urban expansion has diminishing effects on agricultural lands in developed countries, which may alter future agricultural production and food consumption. The heterogeneous effects across countries demonstrate the need for domestic context, including cultural and historical factors, in assessing forest decline, agricultural expansion, and land-use intensity. The co-benefits of Reducing Emissions from Deforestation and Forest Degradation (REDD+) in developing economies are crucial to mitigating emissions while improving forest-dependent livelihoods.

## Highlights

• Forest lands exhibit explosive behavior with a structural decline in several countries
• The magnitude of emissions, forestry, and land use are heterogeneous across economies
• GHG emissions have transboundary tendencies through trade in agriculture and forestry
• Sustained economic productivity declines global emissions and land-use intensity

## Graphical abstract

**Figure:**
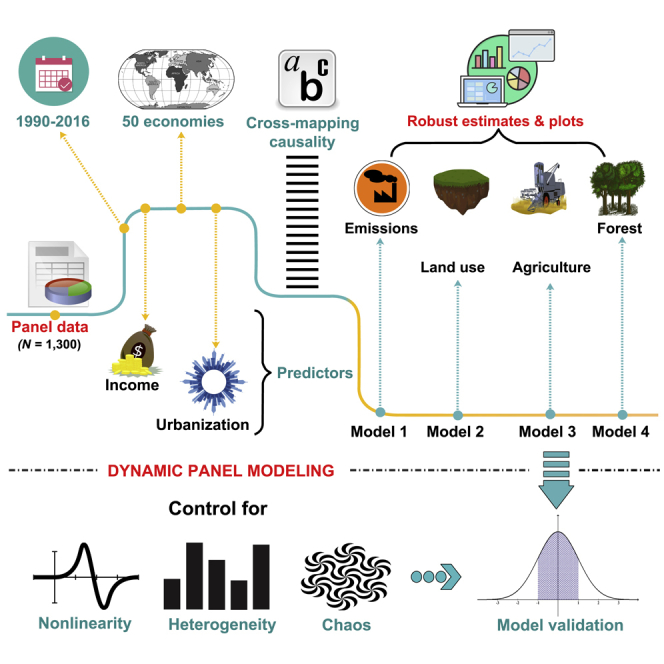

## Introduction

Global land-use intensity is a crucial human cause of land degradation, which may pose threat to ecosystem vitality, leading to loss of natural habitat and changes in landscape (DiSano, 2002; Shukla et al., 2019). Unsustainable land use affects ecological composition including productive lands for forestry and agriculture, which has long-term negative impacts on biodiversity and emissions. Although the global forest cover is improving compared to historical trends decades ago, agricultural expansion, deforestation, and land degradation remain a threat to land conservation in developing countries, especially low-income economies. For example, the global forest area has declined from 32.5% to 30.8% (i.e., 178 million ha) between 1990 and 2020 owing to human-induced changes such as agricultural expansion (FAO, 2020). Although South America observed an unprecedented decline in forest area spanning from 1990 to 2010, Africa witnessed the highest net loss of forest area between 2010 and 2020, whereas the highest net gain between 2010 and 2020 occurred in Asia (FAO, 2020). Climate change mitigation and adaptation in agriculture, forestry, and land use are intertwined via feedback mechanisms, synergies, and trade-offs (Krystal Crumpler et al., 2021; Smith and Olesen, 2010). This implies sustainable land-use management (agroforestry, land-based mitigation options, and integrated landscape approach) is a key adaptation measure to reduce anthropogenic emissions and climate change vulnerability (Hosonuma et al., 2012; Rosenzweig and Tubiello, 2007; Verchot et al., 2007). However, the trilemma existing among agricultural land expansion, forestry, and GHG emissions is driven by population growth, economic development, and urbanization (Shukla et al., 2019). The global population is increasing with increasing demand for food and resources for economic benefit, yet conservation practices require sustainable forest management to limit the rising levels of emissions. The complex nexus between climate change, socio-economic and ecological systems require attention owing to the threat of climate change and its impacts on sustainable development (Denton et al., 2014).

While the literature has reported spatial-temporal trends of ecological portfolio, and ecological resources embodied in trade (Hoang and Kanemoto, 2021), no study has comprehensively assessed the symbiotic relationships existing among land-use intensity, demo-economics, and changes in emission levels. Understanding these dynamic relationships are crucial to unearth historical trends useful to develop conceptual tools for climate change adaptation and mitigation of climate vulnerability. Second, country-specific, regional, and other global crises including the recent COVID-19 pandemic, and economic recessions affected business-*as*-usual, which shifted production and consumption, leading to explosive behaviors across countries. The term “*explosive behavior*” entails unusual trends observed among socio-economic and environmental variables across time. These episodes that capture extremes are indicative of climate change and land-use intensity. Besides, this explains unusual events in emission patterns, resources, and biodiversity exploitation (deforestation, land degradation, ecological footprint, and domestic material consumption) that often contradict existing fundamental patterns. Yet, global multi-regional input-output (MRIO) models may fail to capture explosive behaviors that are significant to tilt the balance between production and consumption.

Here, we ask the following research questions using 27 years of data: (a) what are the drivers of global anthropogenic emissions and land-use intensity? (b) what are the feedback mechanisms, synergies, and trade-offs that underpin emission reduction from agricultural land, forestry, and land use? (c) What are the current trends of ecosystem dynamics across countries (identifying winners and losers)? We use novel econometric techniques to examine global symbiotic relationships, and date-stamping explosive behaviors existing between land-use intensity, demo-economics, and changes in emissions (See model estimation). Using dynamic panel models that capture cross-section dependence, heterogeneity, nonlinearity, and chaotic functions account for the complexities of climate change across countries and income groups. For example, we employ the convergent cross-mapping technique to assess symbiotic relationships while accounting for complex dynamics among variables (See model estimation). Besides, this study for the first time applies the backward supremum right-tail augmented Dickey-Fuller unit root technique based on recursive window widths to control for such unusual behaviors in demo-economic and ecological variables while data-stamping episodes (Baum and Otero, 2021; Phillips et al., 2011). Our study identified episodes of explosive behavior highlighting country-specific events of influx or excesses in emissions, land-use intensity, urban sprawl, and income. The date-stamping explosive behaviors are examined for the top three low-performing and high-performing countries, and subsequently, validated using the US scenario. We opine that these unusual periods of extremely low or high trends could have been triggered by country-specific economic structure and disparities in income distribution.

## Results

### Current trends of ecosystem dynamics

**Figure 1:**
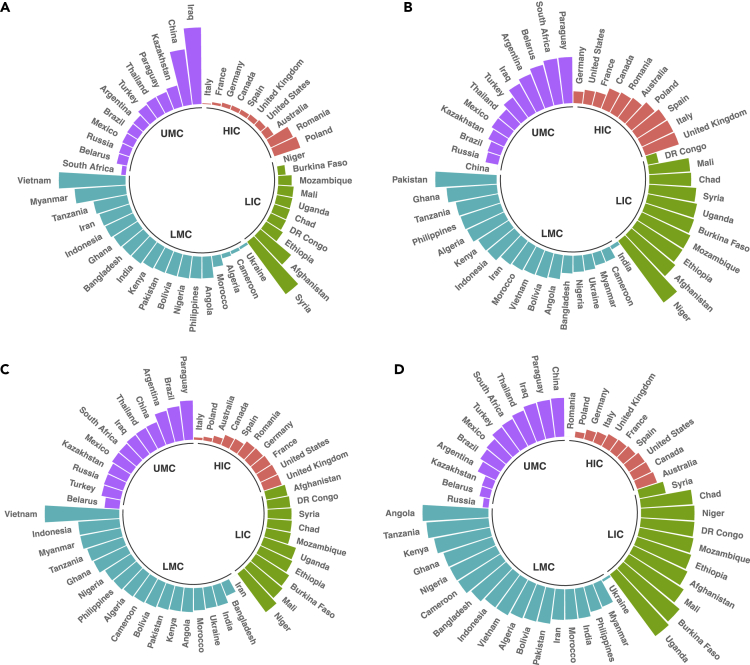
Country-specific average change from 1990 to 2016 (A) income (B) GHG emissions (C) Agricultural land-use (D) urbanization LIC, LMC, UMC, and HIC represent low-income countries, lower-middle-income countries, upper-middle-income countries, and high-income countries. We use a normalization scale [0, 100] to develop country-specific scores from average changes over the sampled time period.

To assess performance, we use a normalization scale [0, 100] to develop country-specific scores from average changes in sampled variables over the 27-year period. For comparison, we categorize performance scores of countries based on income groups (Figure 1). Although average income level improved (between 0.13 and 2.25%) in all economies regardless of income group, Iraq, an upper-middle-income country in the Middle East & North Africa observed the highest gain in income by 2.25%. Niger, a low-income country in Sub-Saharan Africa observed the lowest increase by 0.13%. GHG emissions witnessed an increase in developing countries typically low-income economies. Niger, Pakistan, Afghanistan, Ethiopia, and Mozambique are the top five hotspot countries with rising anthropogenic emissions by 1–1.97% (score = 70.10–100). In contrast, China, India, DR Congo, Germany, and Cameroon saw an average decline in yearly GHG emissions by 0.49–0.97% (score = 0–16.05). The yearly expansion in agricultural land use by 0.24–0.72% (score = 52.92–100) can be observed in low-income and lower-middle-income economies in East Asia & the Pacific, and Sub-Saharan Africa. The top five gainers in agricultural land include Vietnam, Niger, Mali, Indonesia, and Myanmar, whereas the top five losers (i.e., declined by 0.15–0.30%) include Canada, Australia, Poland, Italy, and Iran. The yearly average urban population grew in almost all countries except Russia, and Poland with stabilized growth (0%), whereas Ukraine, and Romania declined by 0.02–0.04%. Conspicuously, the rate of urban population growth was higher in Sub-Saharan Africa, occupying the top five hotspots (i.e., Uganda, Burkina Faso, Angola, Mali, and Tanzania), and 7–15 countries. Yet, countries (specifically high-income economies) with low urban population growth are located in North America, Europe, and Central Asia. The top five countries that saw potential deforestation, viz. decline in forest area by 0.9–2.39% include Mali, Uganda, Nigeria, Algeria, and Pakistan, whereas Niger, Syria, Vietnam, China, and Iran observed yearly average improvement/expansion in forest area by 0.34–61.09% (Figure S1). Niger is singled out in Figure S1 owing to potential explosive behavior observed over the time period. Although historical trends show a decline in forest area, average yearly change reports otherwise, owing to unusual decline in 2005 by 106% and sudden rebound effect by 1,780.7% in 2006, hence, showing a conspicuous behavior requiring attention. The high rate of deforestation (i.e., a decline in forest area) in low-income economies is driven by poverty, high demand for forestry products, and resources to meet energy demands for cooking and heating purposes (Koop and Tole, 2001). Fuelwood charcoal and timber logging are reported as the main determinants of forest degradation in Africa, whereas timber logging is the primary driver of forest degradation in subtropical Asia and Latin America (Hosonuma et al., 2012). Besides, agrarian economies often exploit forest resources through legal or illegal trade to improve economic productivity, especially among poor communities whose livelihood depends on. For example, illegal logging of wood, specifically extinction species such as rosewood has become popular in sub-Saharan Africa owing to its high price value and demand in international markets (Barrett et al., 2010). Thus, these activities serve as a conduit for the spillover of emissions and deforestation embodied in trade (Hoang and Kanemoto, 2021). In contrast, high-income countries are mostly high-tech and service-based economies, hence, depend less on environmental capital including forestry products (Ewers, 2006).

### Date-stamping explosive behavior

**Figure 2:**
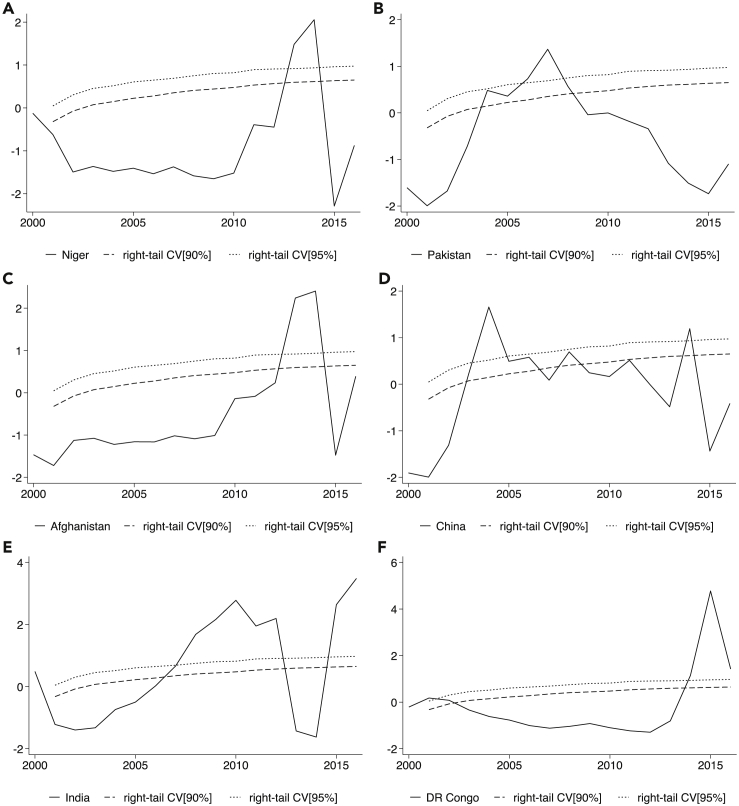
Date-stamping explosive behavior of GHG emissions in top three low-performing and high-performing countries using BSADF test (A) Niger (B) Pakistan (C) Afghanistan (D) China (E) India (F) DR Congo Episodes of explosive behavior occur in 2013–2014 (Niger), 2006–2007 (Pakistan), 2013–2014 (Afghanistan), 2004, 2014 (China), 2008–2012, 2015–2016 (India), and 2001, 2014–2016 (DR Congo). Explosive behaviors are assessed using the backward supremum ADF (BSADF) test based on recursive window widths for data-stamping of episodes.

The unusual trend observed among variables across time periods reveals the existence of dynamic properties requiring further estimation. Explosive behavior of economic indicators often trickle-down to socio-demographic and environmental variables during distress or crises. Thus, explosive behavior may cause sampled variables to deviate from their fundamentals leading to “bubbles.” To account for this unusual behavior, we use the novel backward supremum right-tail augmented Dickey-Fuller unit root technique based on recursive window widths for data-stamping of episodes (Baum and Otero, 2021; Phillips et al., 2011). The estimation technique is applied to the top three low-performing and high-performing countries of sampled variables (i.e., GHG emissions, forest, and agricultural land) to examine for potential explosive behaviors (Figures 2, S2, and S3). We observe a rejection of the null hypothesis of unit root corresponding to the right-tail 90–95% confidence interval, implying the existence of varying periods of explosive behavior across the sampled countries. In Figure 2, one episode of explosive behavior in GHG emissions is observed in Niger (2013–2014), Pakistan (2006–2007), and Afghanistan (2013–2014), whereas two episodes are detected in China (2004, 2014), India (2008–2012, 2015–2016), and DR Congo (2001, 2014–2016). The unusual rebound effect of forest expansion detected in Niger is corroborated by two episodes of explosive behavior occurring in 2001, 2009–2016 (Figure S2). However, no evidence of explosive behavior is found for Nigeria and Syria in the forest model (Figure S2) and Iran in the agricultural land model (Figure S3). Similar episodes of explosive behavior are confirmed among sampled variables using the US as a benchmark (Figure S4). The validation of explosive behavior of sampled variables across the top three low-performing and high-performing countries is suggestive of heterogeneous and/or nonlinear behavior driven by unobserved factors. This infers the adoption of business-*as*-usual estimation techniques for variables exhibiting sensitive behaviors may be erroneous. The identified episodes of explosive behavior highlight country-specific events of influx or excesses in emissions, land-use intensity, urban sprawl, and income. These unusual periods of extremely low or high trends could have been triggered by country-specific or global financial crises.

### Assessment of symbiotic relationships

**Figure 3:**
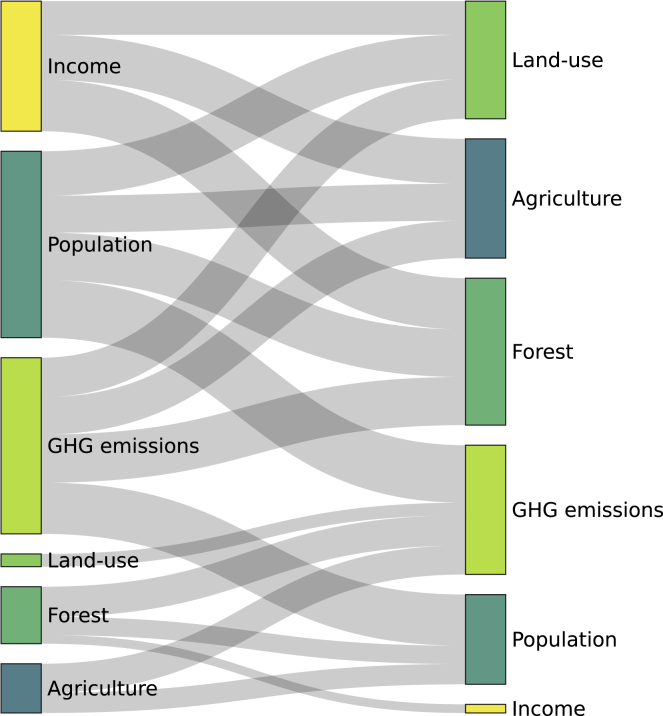
Symbiotic relationship among sampled variables across countries The parameters were estimated using the convergent cross-mapping technique to examine causal effects. The Sankey diagram presented shows the predictor (left) to target (right) causal relationship. We only presented causal effect relationships that are statistically significant. The arrow represents the causal links with width proportionate to the weight/coefficient of the flow, whereas the rectangles with corresponding texts are the nodes.

Owing to the limitations of standard empirical techniques to examine dynamic systems with complex, nonlinear, and chaotic functions, we employ the non-parametric convergent cross-mapping (CCM) algorithm (Li et al., 2021) to assess causality by mimicking biological symbiotic relationships. Contrary to standard econometric techniques that predict outcomes using causes, the CCM algorithm employs the reverse—arguing that the search for causes, in reality, begins with an outcome to ascertain whether its dynamic structure is embedded with the signature of a cause (Schiff et al., 1996). Additionally, we control for transitivity and external forcing of non-coupled series that exist in ecological systems. Thus, the CCM models presented herein account for complexities that are problematic in panel literature that examine causations (Sugihara et al., 2012). The validation of causality infers the paired variables share information about a common dynamic system that underpins the direction of causality (Sugihara et al., 2012). The Sankey diagram presented in Figure 3 shows statistically significant causal networks among sampled variables. The unidirectional coupling observed from income to land-use, income to agriculture, and population to land-use shows commensal or amensal relationships that have policy implications. Previous studies on another scope classify the unidirectional causality between paired variables as either conservation or growth hypotheses synonymous with commensal or amensal relationships (Ozturk, 2010). The conservation hypothesis posits that sustainable productivity is driven by long-term resource utilization. In contrast, the growth hypothesis postulates that natural resource utilization is driven by economic productivity. This confirms the effect of urban population on land-use, and the influence of income on land-use, and agriculture. In contrast, the bidirectional coupling is validated between GHG emissions and land-use, GHG emissions and forest, GHG emissions and urban population, GHG emissions and agricultural land, agricultural land and urban population, income and forest, and forest and population. The bidirectional coupling aka feedback hypothesis postulates a long-term mutual relationship between natural resource utilization and economic development. These mutual relationships validate known feedback mechanisms in biological systems where organisms are co-dependent.

### Predictors of changes in land use

**Figure 4:**
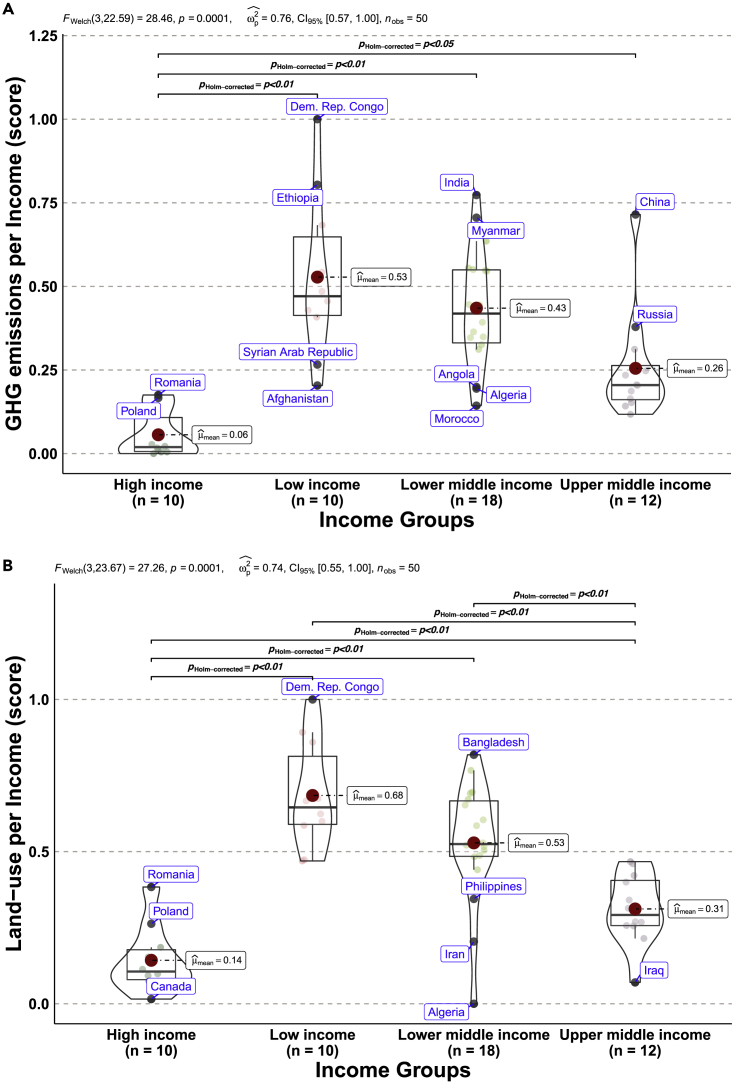
Distribution across income groups (A) GHG emissions per Income (B) Land-use intensity per Income The pairwise test using the Games-Howell technique shows only statistically significant comparisons. () represents the within mean across income groups. The output of the frequentist analysis *F*_*welch*_ (.) = #, *p* = #, $\hat{\omega_{p}^{2}}$ = #, CI_95%_ [#, #], *n*_*obs*_ = # denote the parameter test statistic, significance of the *p*-value, estimate of the effect size, confidence interval, and number of observations.

**Figure 5:**
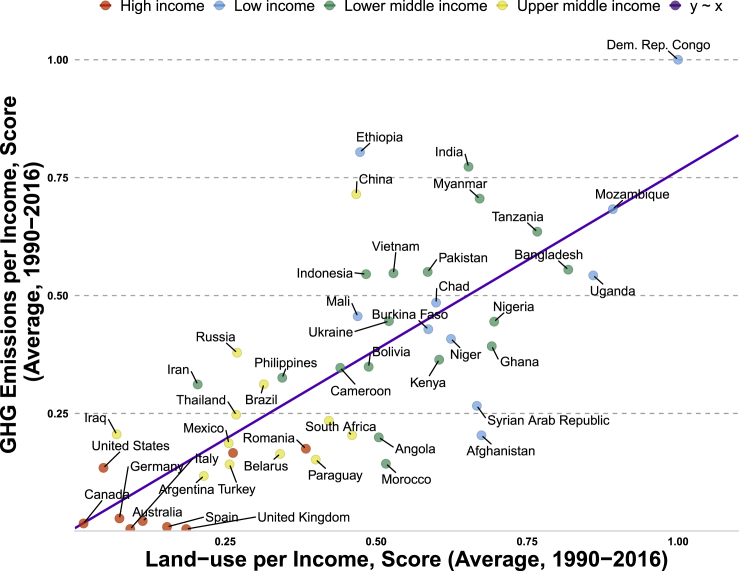
Relationship between GHG emissions per Income and land-use intensity per Income across income groups Both population and income dynamics are accounted for in both variables, hence, showing a positive monotonic relationship that validates the feedback coupling mechanism of GHG emissions and land use in the convergent cross-mapping causality.

Accounting for decadal effects of income level on land-use intensity and anthropogenic GHG emissions using Games-Howell pairwise test (Patil, 2021) across income groups provides statistically significant between-group comparisons. Based on Welch’s parametric one-way ANOVA hypothesis testing, several country-specific means are compared without restrictions on equal sample variances (Welch, 1951). In the output distribution plots, we use outlier tagging to detect extremely high and low performance across income groups. A visual inspection of Figure 4 confirms the statistical evidence showing the ranking: low-income > lower-middle-income > upper-middle-income > high-income. This infers the within-mean of low-income countries is higher than their counterparts in both emissions and land-use models. Another observation is that economies with low-income levels have higher GHG emissions and land-use intensity, whereas high-income countries exhibit low emissions and land-use intensity. For example, DR Congo produces higher GHG emissions per income, whereas Afghanistan is the lowest emitter per income in low-income countries (Figure 4A). Countries with low-income levels often depend on vintage technologies for agriculture, forestry, and land use, with little or no sustainable practices and environmental consciousness—which coincides with a pollution-driven growth trajectory at the early stages of economic development (Sarkodie and Strezov, 2019). In contrast, Romania exhibits the highest land-use intensity per income, whereas Canada is the lowest land-use per income economy in high-income countries (Figure 4B). To further strengthen the argument, Figure 5 examines the relationship between GHG emissions and land use by accounting for both population and income dynamics. The resultant nexus shows a positive monotonic relationship that validates the distribution plot and feedback coupling mechanism of GHG emissions and land use in the convergent cross-mapping causality. In a similar ranking, while high-income economies are associated with low emissions and land-use intensity, low-income economies including inter alia, DR Congo, Mozambique, and Uganda have close linkage with high land-use intensity and GHG emissions (Figure 5). In another scenario (Figure S5), the effect of urban population on land-use intensity is glaring, showing that income group with high urban population has lower land-use intensity, whereas countries with low urban population rate have higher land-use intensity. A similar study found little impact of urban expansion on land-use intensity, viz. forest degradation in Africa and Latin America, yet, the impact is high in Asia (Hosonuma et al., 2012). Noticeably, the lowest change in agricultural land in low-income countries far exceeds the highest agricultural land change in high-income economies. This describes potential diminishing effects of agricultural land in developed countries, altering agricultural production.

### Drivers of anthropogenic emissions and land use

**Figure 6:**
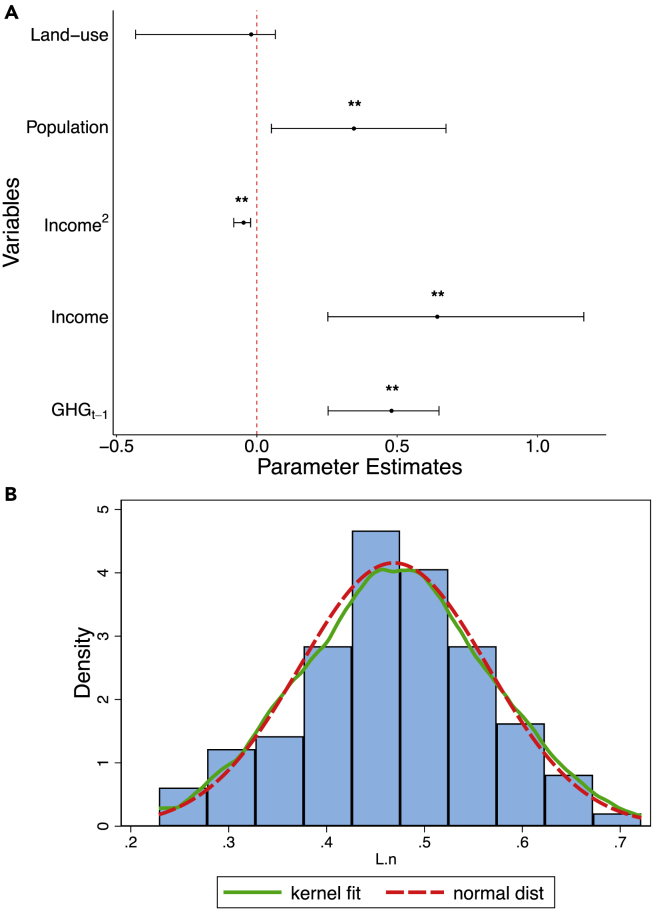
Parameter estimation (A) GHG emissions, income, and land-use (B) Model validation using bootstrap distribution for all autoregressive coefficients A visual inspection of the histogram shows the bootstrap-simulated distribution is normally distributed, which is informative for investigating residual stationarity. The parameter estimates of all variables excluding land use are statistically significant at *p-value* < 0.05 (**∗∗**). The heterogeneous slope testing—Standard delta test $\tilde{\Delta }$ (9.030, *p< 0.01*), adjusted delta test ${\tilde{\Delta }}_{adj}$ (10.240, *p< 0.01*), and HAC robust delta test ${\tilde{\Delta }}_{HAC}$ (3.664, *p< 0.01*), confirms heterogeneous effects across countries. We used bootstrap corrected dynamic FE regression (n = 1300) based on cross-section dependence resampling and analytical heterogeneous initialization to achieve convergence. The estimated model has bootstrapped standard errors, bootstrap 95% (percentile-based) confidence intervals, and statistical inferences performed with non-parametric bootstrap. Residual diagnostics: CD-test (−0.23) & *p*-*value* (0.821); Pesaran’s CADF test (−1.375) & *p*-*value* (0.998).

To examine relationships that identify determinants of GHG emissions, agriculture, forestry, and land use, we adopt panel dynamic estimation techniques that investigate global common shocks, spillover effects, heterogeneous effects, and controls for both endogeneity and omitted-variable bias. We use the panel bootstrap corrected fixed-effects regression based on cross-section dependence resampling and analytical heterogeneous initialization to achieve convergence (De Vos et al., 2015). We observe significant (*p-value* < 0.01) inertia effects in historical anthropogenic emissions that predict the future rise in GHGs (Figures 6 and S6). This explains why 60% (30/50) of the sampled countries including 11 of 15 Sub-Saharan African economies show a positive yearly average in emission levels. Owing to the absorptive capacity of forests and crops, rising levels of anthropogenic GHG emissions significantly (*p-value* < 0.05) trigger global expansion of land use for agricultural and forestry activities (Figure S7). We find evidence of a global shift from forestry to agricultural land use, which may have been triggered by the increasing global demand for food to control threats of food insecurity––that permeates many low-income economies. Our model provides statistically significant (*p-value* < 0.05) evidence supporting the escalation effect of urban population on GHG emissions, land use (Figure S7), and agricultural land (Figure S8A), but has mitigating effects on forest land use (Figure S8B). Although urbanization is a threat to future land allocation, we identify opportunities for reducing forest loss with improved innovation and technology. The yearly fixed-effects of several countries excluding Kazakhstan and Niger predict (*p-value* < 0.01) future forest expansion as innovation increases over time (Figure S9). Such predicted threat of forest loss aside from low forest cover in Kazakhstan may be linked to the failure to address climate change in forest policies (Sehring, 2012). The biological diversity loss of forests in Niger can be associated with degradation owing to agricultural expansion, inadequate forest management, immature harvesting of forest products, and climate change-driven desertification and wildfires (WA BiCC, 2020).

Growth in income level exhibits insignificant positive effect on forest, but insignificant negative effect on land-use intensity. Contrary, income growth significantly (*p-value* < 0.01) spurs GHG emissions and agricultural land use; however, the coefficient (*p-value* < 0.01) on the quadratic of income is negative in both emission and agricultural land-use models. This implies that income level exhibits a parabolic shape, hence, has diminishing effects on GHG emissions and agricultural land use. From the estimated slope relationship, 1% growth in income exacerbates GHG emissions by 0.74% and agricultural land use by 0.12%. Using the approximation $\left[{\hat{\beta }}_{1}GDP/\left(-2{\hat{\beta }}_{2}GDP^{2}\right)\right]$, the turning points for both models are calculated as 6.912 (in *log*) for the GHG model and 7.333 (in *log*) for the agricultural land-use model. This infers the return to income level becomes zero at ∼ US$1005 per capita in the GHG model and ∼US$1530 per capita for the agricultural land-use model. This has policy implications as income data shows about 64% of countries have average income levels above the turning point in the GHG model, whereas 52% of economies are beyond the extremum point in the agricultural land-use model. Our model reveals the possibility of income level increasing anthropogenic GHG emissions until it reaches an extremum point of U$1005 per capita and declines thereafter. Although the seemingly low turning points may have been influenced by the dominance of low- and lower-middle-income countries sampled in the model, many of these countries are still below the extremum point. This describes extreme income inequality where a large population in low-income countries are poor, hence, averaging income level affect the few wealthy population. Countries with average income below the turning point in the GHG model include Nigeria, Afghanistan, Pakistan, India, Ghana, Kenya, Vietnam, Bangladesh, Myanmar, Mali, Chad, Tanzania, Burkina Faso, Uganda, Mozambique, Niger, DR Congo, and Ethiopia. Similarly, income growth escalates agricultural land-use intensity until a turning point of U$1530 per capita before declining. Other countries below the extremum point in the agricultural land-use model including the listed economies in the GHG model comprise Indonesia, the Philippines, Angola, Syria, Bolivia, and Cameroon.

## Discussion

This study examines ecosystem dynamics to better understand the historical trends and performance of nations. We further date-stamped episodes of analyzed explosive behavior for unusual trends observed among sampled variables. The convergent cross-mapping for causations confirmed bidirectional coupling, both conservation and growth hypotheses among a network of variables, specifically, between natural resource utilization and economic productivity. First, the coupling effect between environmental footprints (i.e., including land use, agriculture, and forest resources) and growth implies the institutionalization of environmentally friendly policies that decline resource intensity will affect sustained economic development and vice versa. In contrast, the coupling effect with limited green growth has implications on climate change and its impacts. Because of the feedback effect of resource consumption and economic development, production often increases to meet consumption demands triggered by economic activities. Although decoupling natural resources from economic development appears useful in resource-intensive and carbonized economies, the coupling effect of economic development and resource sustainability (including the adoption of artificially engineered resources) could be more practical to achieve sustainable economic development. Second, the conservation hypothesis infers ecological infrastructure of a country determines the composition of the economic pathway (i.e., circular or linear economy). The conservation hypothesis supports the notion of eco-sufficiency—where environmental footprints such as land use, agriculture, and forest resources decline through sustainable production and utilization of services (Princen, 2005). Existing literature argues that the introduction and adoption of conservation and management (typically in energy and production sectors) options to reduce environmental degradation may hinder sustainable economic development (Tallis et al., 2008). In contrast to this notion, conservation may not always thwart economic productivity if the natural resource portfolio and production are efficiently diversified using modern technologies and innovations such as eco-metamaterials (Rosemarie and Michael, 2022). This implies that resource efficiency and eco-sufficiency can be achieved while meeting immediate demands for economic activities. In this scenario, countries can shift from a brown economic pathway to green economic growth. In a contrary scenario, the growth hypothesis is useful in examining healthy economic pathways—by accounting for both resource intensity and efficiency. Natural resource intensity entails resources required per unit of economic productivity. High resource intensity represents inefficient growth-resource interaction where high resources and environmental costs underpin economic activities. However, low resource intensity represents efficient growth-resource interaction where there is low resource exploitation and utilization, hence, reducing environmental externalities. Natural resource efficiency occurs when resource requirement per economic productivity declines owing to stringent environmental policies, enhanced technology, and sustainable infrastructures. Thus, the composition of the economic structure determines the production and consumption of natural resources, viz. environmental footprints such as land use, agriculture, and forest resources.

The validation of potential spillover effects across countries implies GHG emissions have transboundary tendencies through trade in agricultural and forestry products that affect land-use intensity, especially in low-income countries. However, the magnitude of anthropogenic emissions, forestry, and agricultural land use appears heterogeneous across income groups. For example, fossil fuels dominate the energy portfolio of developed economies driving global emissions owing to the increase in production and consumption (Le Quéré et al., 2019; Raupach et al., 2007; Sarkodie, 2022). However, high adaptation readiness and mitigation options in developed economies increase climate resilience, hence, reducing the effects of GHG emissions compared to developing economies (Sarkodie et al., 2022). Although economic productivity has improved across countries, there is evidence of outgrowth in anthropogenic GHG emissions in developing countries, specifically in low-income economies. The turning point of income in both quadratic models shows GHG emissions and agricultural land-use intensity across high-income and upper-middle-income countries have lessened at some point but somewhat unclear if this decline occurred around US$1005–1530 per capita. Nevertheless, we still found structural evidence confirming countries with low average income are characterized by high GHG emissions and high land-use intensity, whereas emissions and land-use intensity diminish as income increases. This parabolic shape confirms the existence of the environmental Kuznets curve hypothesis, which posits income outgrowth characterized by extensive resource utilization, pollution, and waste intensity at developmental stages in weakly regulated countries. However, emissions levels, waste, and resource intensity decline after realizing a specific turning point of income in stringent and regulated countries with environmental awareness (Dasgupta et al., 2002; Sarkodie and Strezov, 2019). Although income growth is not an exclusive determinant of anthropogenic emissions and land-use intensity, the fundamental difference between income groups in terms of production and consumption patterns is determined by income distribution. Similarly, income level underpins the dynamics of agriculture, forestry, and land-use intensity (FAO, 2022).

Our date-stamping technique shows explosive behavior for forest lands, with many countries observing a structural decline in forest areas. Deforestation is reportedly increasing and becoming a global threat owing to the decline in forest areas embodied in global supply chains (Hoang and Kanemoto, 2021). Studies have reported a link between deforestation and agricultural productivity. Deforestation affects water resources and agricultural productivity by declining pasture productivity, livestock production, and crop productivity (Lawrence and Vandecar, 2015). Thus, large-scale conversion of forests in one region could have spillover effects that hamper sustainable food production (Gordon Line et al., 2005; Knoke et al., 2013). The historical changes in forest area can be attributed to deforestation owing to an increase in commodity demand, a shift from forestry to agriculture—especially when food security is a threat, urbanization-driven infrastructure expansion, and wildfires (Curtis et al., 2018). Expansion of agricultural land remains the primary driver of forest degradation and deforestation, yet the resilience of food production systems and their adaptive capacity to future changes depend on forest biological diversity (FAO, 2022). Agricultural expansion is evident in countries, typically developing economies that depend heavily on agriculture to meet economic targets. For example, while subsistence agriculture is the main driver of deforestation in Africa and subtropical Asia, large-scale commercial agriculture is the primary determinant of deforestation in Latin America (Hosonuma et al., 2012). The concept of scale effect applies here, given the expansion in agricultural land resources for productive use to meet the growing population and global demand for food and domestic material resources for global supply chains. This explains why anomalies identified in forest land use and agricultural land expansion are mostly located in low-income countries with extreme poverty (FAO, 2022). Although wealthy nations are reported to conserve disappearing forests and embark on further afforestation, low-income nations with little forest cover are reported to likely consume the remaining resources at faster rates than low-income economies with huge forest resources (Ewers, 2006). The presence of heterogeneous effects across countries demonstrates the need for domestic context, viz. cultural and historical factors in assessing agricultural expansion, forest decline, and land-use intensity (FAO, 2022). The interaction between local forces (i.e., cultural values, access to resources, and corruption), regional policies (i.e., trade and environmental policies, institutional quality, and commodity markets), and global processes (i.e., subsidies, global commodity markets, and international agreements) underpin local resources and responses that could determine conservation and management outcomes (Giller et al., 2008). Thus, achieving sustainable development requires tailoring global readiness, adaptation, and mitigation options to the local context and identifying opportunities that decline vulnerabilities and effects of climate change.

### Limitation of the study

Our empirical estimation has limitations that may have affected statistical inferences. First, the land-use indicator consists of arable land, forests, permanent cropland, and pasture but excludes built-up areas and others, which may affect the ability to capture changes in land distribution, especially in urbanized countries. However, the adoption of a novel panel heterogeneous technique allows controlling for unobserved heterogeneity and omitted-variable bias. Second, our model doesn’t assess the equilibrium relationship between country-specific supply and demand as in the case of production-side assessment in input-output models, yet, we use econometric models that examine historical patterns and drivers of inputs and outputs useful for policy formulation. Such information is useful to mitigate land-use and emission threats and prevent irreversible damage to natural resources. Besides, we identify opportunities for sustainable land management and land-use planning strategies. For example, we observe that most developing countries are more likely to address the ecological and economic benefits of land use rather than climate change effects. This tradeoff highlights the role of Reducing Emissions from Deforestation and Forest Degradation (REDD+) in developing economies that has co-benefits in mitigating anthropogenic emissions while improving the income and social equity of those whose livelihood depends on forestry (Denton et al., 2014). Extending the forest carbon partnership to include more developing countries would help in building REDD + readiness, hence, has a long-term impact on forest carbon stock conservation, sustainable forest management, and emission reduction from forest degradation and deforestation (FCPC, 2022).

## Ethical approval

This article does not contain any studies with human participants performed by any of the authors.

## Informed consent

This article does not contain any studies with human participants performed by any of the authors.

## STAR★Methods

### Key resources table

**Table:** 

REAGENT or RESOURCE	SOURCE	IDENTIFIER
**Deposited data**		

World Development Indicators	World Bank national accounts data, and OECD National Accounts data files.	https://data.worldbank.org/indicator

**Software and algorithms**		

Stata 16	StataCorp LLC	https://www.stata.com/
Rstudio 2022.02.3 Build 492	RStudio, PBC	https://www.rstudio.com/

### Resource availability

#### Lead contact

Further information and requests for resources should be directed to and will be fulfilled by the lead contact, Samuel Asumadu Sarkodie (asumadusarkodiesamuel@yahoo.com).

#### Materials availability

This study did not generate new unique reagents.

### Method details

#### Data

The empirical assessment is based on over decadal (1990–2016) data derived from the World Bank database (World Bank, 2020) consisting of 50 countries in 7 regions [i.e., East Asia & Pacific ( 7 economies), Europe & Central Asia (12 economies), Latin America & Caribbean (5 economies), Middle East & North Africa (5 economies), North America (2 economies), South Asia (4 economies), and Sub-Saharan Africa (15 economies)]. The sampled data comprises anthropogenic GHG emissions, income level, urban population, agricultural land, and forest area (used as a proxy for forest land use). The adoption of GHG emissions as an indicator of environmental vitality enables the assessment of the direct effect of global emission status on climate change. While GDP per capita is used as an indicator of income level, urban population is used to examine the role of urbanization on changes in land resources. Agricultural land used in this study captures cropland, arable land, and permanent pasture whereas forest area is the proportion of land covered by forests. The indicator used to comprehensively assess changes in land use (*LU*) is constructed using the weights (*W*_*A*_, *W*_*F*_) of both agricultural land (*A*) and forest area (*F*) expressed as:

(Equation 1) $$LU=\left(A*W_{A}+F*W_{F}\right)/2,\;W_{A}=\frac{A}{A\;+\;F}\;\text{and}\;W_{F}=\frac{F}{A\;+\;F}\text{,}$$

Multiple data transformations and quantifications including logarithm, normalization, first-difference, and means were used to capture specific data features in the models. We quantified low- and high-performing countries across income groups using the average percentage change in sampled variables over time. The graphical relationship between GHG emissions and land-use intensity was investigated across income groups while accounting for both population and income dynamics (Figure 6). Both variables were divided by income level and subsequently averaged over the sample period before normalization to generate country-specific scores using the expression: score (0,1) = [Vi-V_min_]/[V_max_-V_min_], where V_min_ represents the minimum data point whereas V_max_ denotes the maximum data point.

#### Model estimation

To visualize the distribution across income groups, we used the Games-Howell test (i.e., parametric technique with no equal variance but normally distributed residuals) for the between-group pairwise comparison (Pohlert, 2014). The visualization produces detailed statistical inferences (Patil, 2021) based on Welch’s one-way ANOVA (parametric technique) hypothesis testing procedure with parametric effect size estimation (Welch, 1951). Assessing unexplained characteristics of historical data across countries is a useful step in econometric modeling. Thus, the unusual characteristics observed among variables across time periods reveal the presence of dynamic properties requiring attention. Explosive behaviors in economic indicators have a trickle-down effect on demographic and ecological markers during crises. From a policy perspective, explosive behaviors may cause historical trends to deviate from their fundamentals leading to unusual and unexplained scenarios. Our empirical analysis accounted for such unusual behaviors in demo-economic and ecological variables using the backward supremum right-tail augmented Dickey-Fuller unit root technique based on recursive window widths for data-stamping of episodes (Baum and Otero, 2021; Phillips et al., 2011). The date-stamping explosive behaviors of demo-economic and ecological variables were examined for the top 3 low-performing and high-performing countries namely Niger, Pakistan, Afghanistan, China, India, and DR Congo. We further used the dataset of the US to validate the estimated behaviors over the time period.

Global partnerships between countries and across income groups may stimulate spillover effects, pollution-embodied in trade, deforestation-embodied in trade, and land-degradation-embodied in international trade. Besides, economies are prone to global common shocks such as the recent Covid-19 pandemic and other historical global economic recessions. Yet, the impact may be heterogeneous across economies depending on the economic structure and ecological status. Beyond the challenges of traditional panel data models, income groups exhibit economic diversification, income disparities between population structures, varying pollution levels, and diverse environmental policies that affect the specification of ecological models. To account for this, we examined panel cross-section dependence (CD) and heterogeneous effects using the Pesaran-CD test (Pesaran, 2004) for both variable and residual diagnostics and standardized Swamey-tests (i.e., Standard delta test $\tilde{\Delta }$, adjusted delta test ${\tilde{\Delta }}_{adj}$, and HAC robust delta test ${\tilde{\Delta }}_{HAC}$) (Pesaran and Yamagata, 2008) for panel slope homogeneity (i.e., a violation of the test implies heterogeneous effects). After confirming panel cross-section dependence and heterogeneous effects, we used the panel unit root test (i.e., CADF is a 2^nd^ generational panel unit root test for heterogeneous panels) to examine stationary properties of sampled variables (Lewandowski, 2006). This technique curtails the possibility of spurious regression while improving model specification. We observed level stationary characteristics for almost all sampled series.

Subsequently, we assessed symbiotic relationships using the convergent cross-mapping technique while accounting for complexities, and dynamics among variables. Contrary to standard panel techniques that fail to report true causality in non-linear dynamic systems, the empirical dynamic modeling technique, viz. convergent cross-mapping solves the challenges of traditional panel methods by predicting causality amidst variables that exhibit nonlinearities, explosive behaviors, and complexities (Li et al., 2021). The convergent cross-mapping is a non-parametric technique where manifolds are reconstructed with one-to-one mapping if, for example, both *GHG* and *Income* variables occur within the same dynamic system with manifold ***M*** (Sugihara et al., 2012). Thus, causality (*GHG → Income*) exists if the reconstructed manifold (***M***_*Income*_) cross-maps *GHG* with accuracy in prediction for *GHG|****M***_*Income*_.

After assessing the causal associations using the convergent cross-mapping method, we proceeded to estimate the determinants of anthropogenic emissions and land use using bootstrap-corrected dynamic fixed-effects regression. For brevity, the generic dynamic panel model can be expressed as (De Vos et al., 2015; Everaert and Pozzi, 2007):

(Equation 2) $$y_{i,t}=\alpha_{1}y_{i,t-1}+\ldots +\alpha_{q}y_{i,t-q}+\beta x_{i,t}+u_{i}+\varepsilon_{i,t}$$

where *y* denotes the dependent variable across countries *i* in time period *t*, *β* is estimated parameters (coefficient vector) of exogenous variables *x*, $\alpha_{1}-\alpha_{q}$ represent autoregressive coefficients of lagged-dependent variables, $u_{i}$ denotes the fixed-effect across countries, and $\varepsilon_{i,t}$ is the observation-specific error across countries over the time period. Using the model specification in Equation 2, we developed four models where the EKC hypothesis is examined using income, quadratic income, urban population, and land use in GHG emission function (Figure 6). Second, we validate the EKC hypothesis using income, quadratic of income, urban population, disaggregated land-use, i.e., forestry and agricultural land in GHG emission function (Figure S6). Third, we assessed the effect of GHG emissions, income, and urban population on land-use intensity (Figure S7). Finally, we examined the impact of GHG emissions, income, quadratic income, urban population, and forestry on agricultural land (Figure S8A).

Advantageously, the bootstrap-corrected dynamic fixed-effects estimator controls for panel cross-sectional dependence and heteroskedasticity patterns that undermine standard correction techniques (Everaert and Pozzi, 2007). The bootstrap-corrected dynamic fixed-effects regression (n = 1300) is improved to incorporate cross-sectional dependence resampling and analytical heterogeneous initialization to achieve convergence (De Vos et al., 2015; Sarkodie and Owusu, 2020). The cross-sectional dependence resampling enforces cross-section-specific error terms but with identical time indices across countries. Besides, the analytical heterogeneous initialization technique is utilized to generate the initial conditions, i.e., multi-variate normal distribution sample with country-specific means and variance-covariance matrices in the resampling procedure (De Vos et al., 2015; Everaert and Pozzi, 2007). The estimated model has bootstrapped standard errors, bootstrap 95% (percentile-based) confidence intervals, and statistical inferences performed with non-parametric bootstrap. The estimated models are further diagnosed for residual independence using bootstrap distribution for all autoregressive coefficients, residual cross-sectional dependence (CD-test), and residual panel stationarity tests (Pesaran’s CADF test).

## Data Availability

Data: This paper analyses existing, publicly available data. These accession numbers for the datasets are listed in the key resources table. Code: This paper does not report original code, however, the code for analysis was written in Stata and R, available from the lead contact upon request. Additional Information: Any additional information required to reanalyze the data reported in this paper is available from the lead contact upon request.
