# Supplementary material for: Global land-use intensity and anthropogenic emissions exhibit symbiotic and explosive behavior
Source: iScience. 2022 Jul 14;25(8):104741. doi: 10.1016/j.isci.2022.104741 (PMC9352532; doi:10.1016/j.isci.2022.104741)
Supplement: Document S1. Figures S1–S9 [file mmc1.pdf]

iScience, Volume *25*

Supplemental information

Global land-use intensity and anthropogenic emissions exhibit symbiotic and explosive behavior

Samuel Asumadu Sarkodie and Phebe Asantewaa Owusu

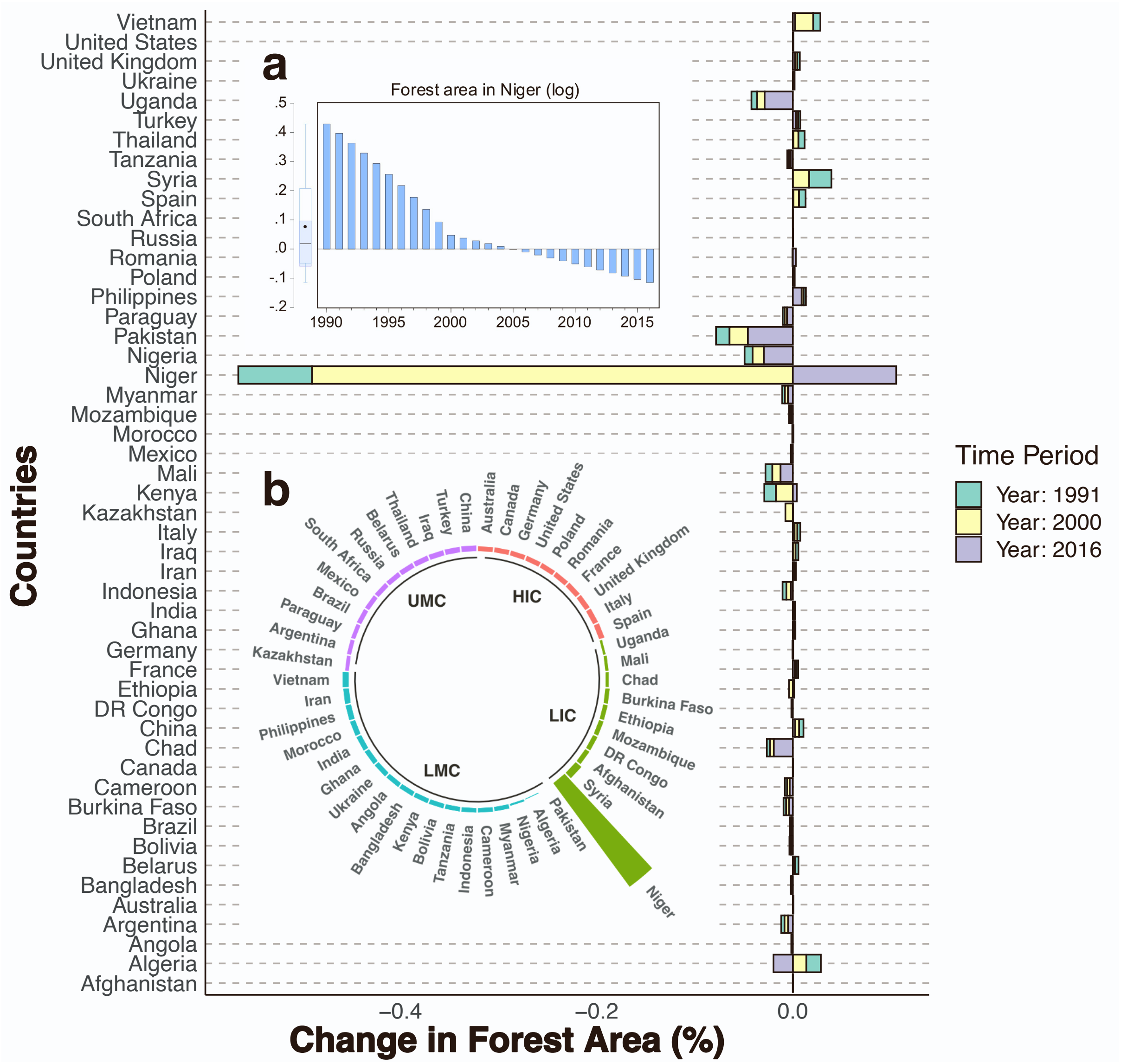

Figure S1. Percentage change in forest area by comparing 1991, 2000, and 2016 time periods., Related to Figure 1.

The % change was calculated using previous years across economies. The inside plot (a) represents the log historical trend of forest area in Niger (b) denotes the country-specific average change in forest area from 1990 to 2016. LIC, LMC, UMC, and HIC represent low-income countries, lower-middle-income countries, upper-middle-income countries, and high-income countries. Niger is singled out due to potential explosive behavior observed over time. While historical trends show a decline in forest area, average yearly change reports otherwise, due to unusual decline in 2005 by 106% and sudden rebound effect by 1,780.7% in 2006, hence, showing a conspicuous behavior requiring attention.

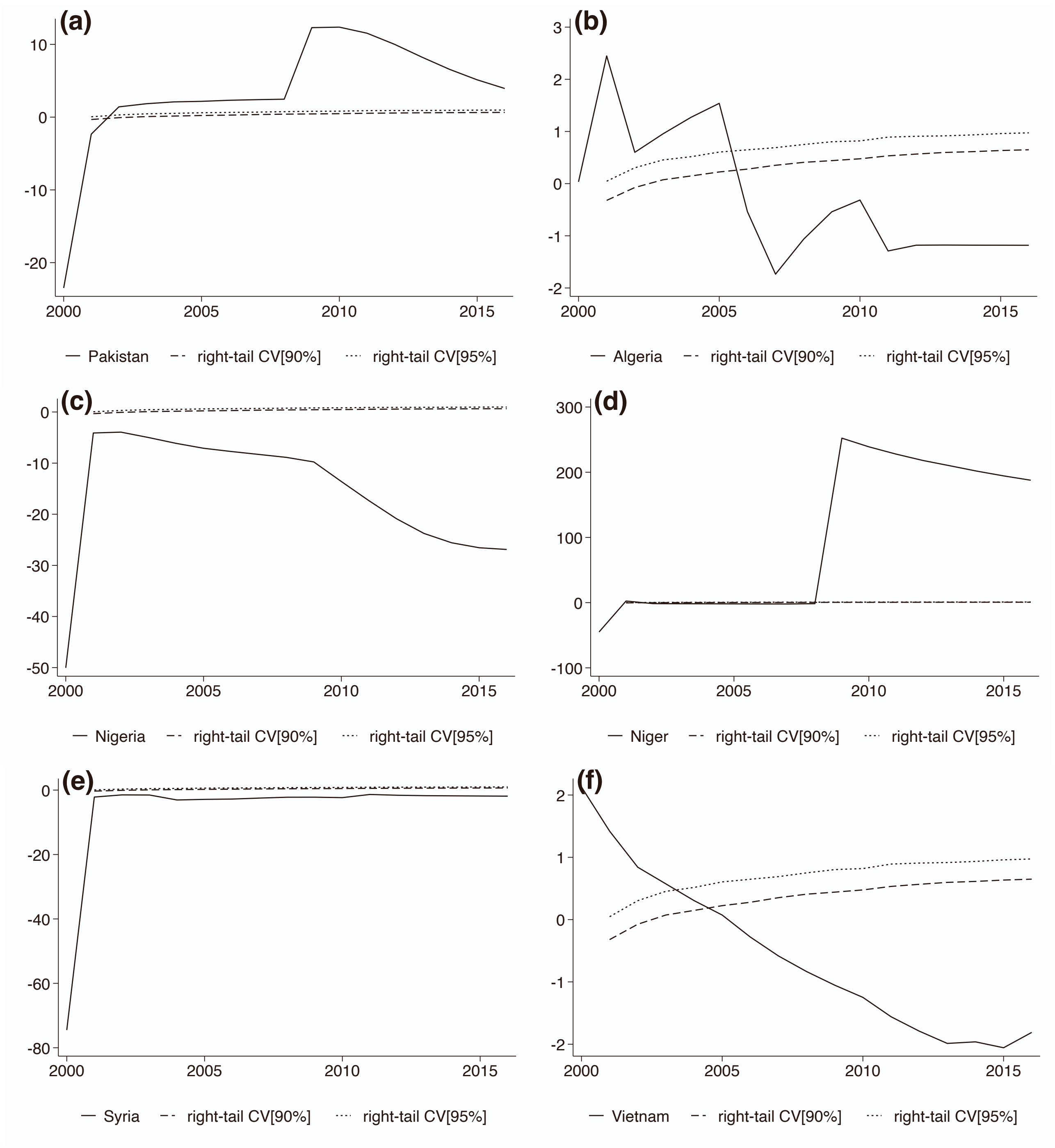

Figure S2. Date-stamping explosive behavior of forest land-use in top 3 low-performing and high-performing countries using BSADF test (a) Pakistan (b) Algeria (c) Nigeria (d) Niger (e) Syria (f) Vietnam. Related to Figure 2.

Episodes of explosive behavior occur in 2002-2016 (Pakistan), 2001-2005 (Algeria), 2001, 2009-2016 (Niger), and 2001-2003 (Vietnam) whereas no episodes of explosive behavior occur in Nigeria and Syria, since the estimated test is insignificant.

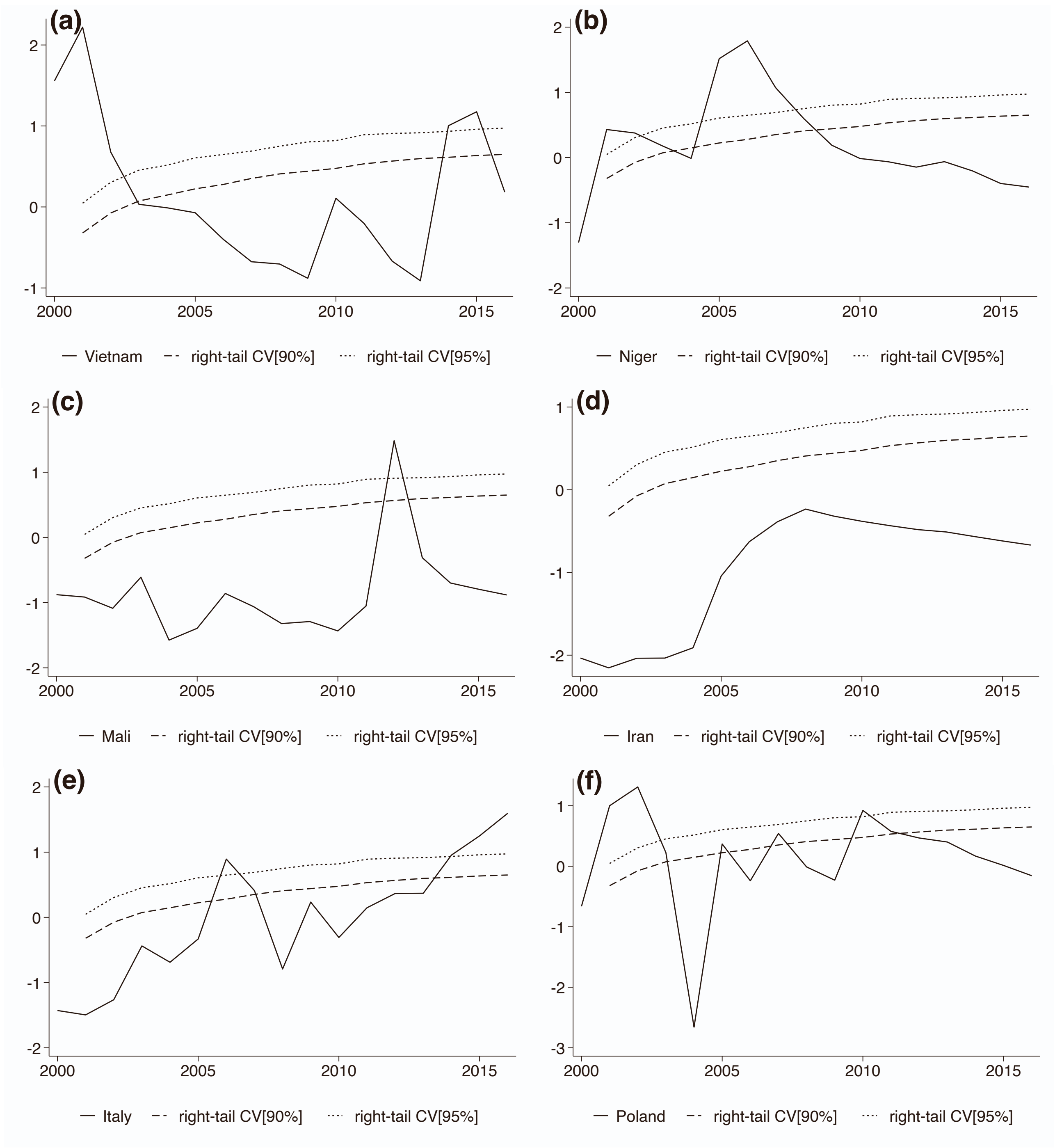

Figure S3. Date-stamping explosive behavior of agricultural land-use in top 3 high-performing and low-performing countries using BSADF test (a) Vietnam (b) Niger (c) Mali (d) Iran (e) Italy (f) Poland. Related to Figure 2.

Episodes of explosive behavior occur in 2001-2002, 2014-2015 (Vietnam), 2001-2002, 2005-2007 (Niger), 2012 (Mali), 2006, 2014-2016 (Italy), and 2001-2002, 2010 (Poland) whereas no episodes of explosive behavior occur in Iran, since the estimated test is insignificant.

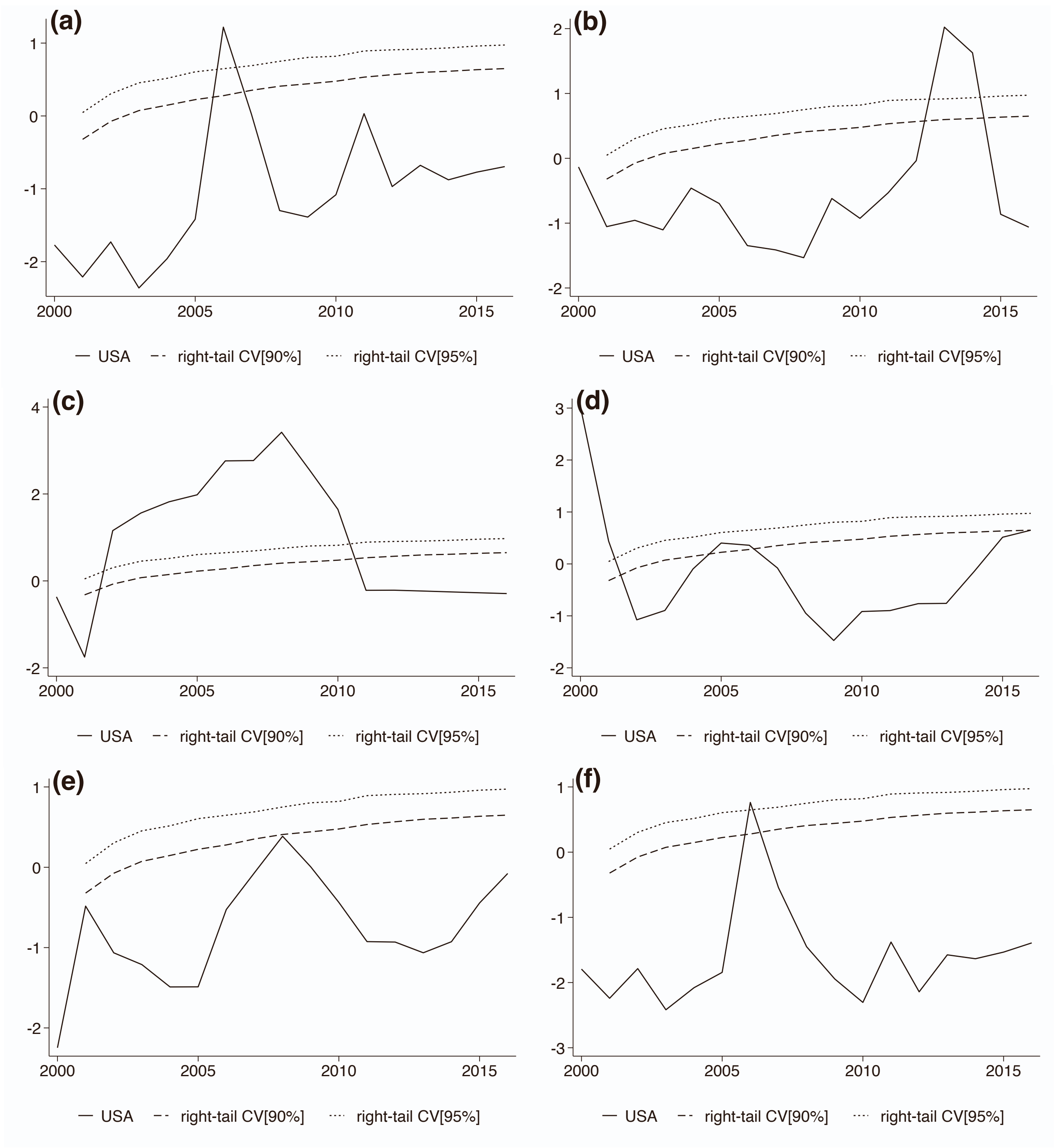

Figure S4. Date-stamping explosive behavior of sampled variables in USA using BSADF test (a) Agricultural land (b) GHG emissions (c) Forest (d) Income (e) Urban population (f) Land use. Related to Figure 2.

Episodes of explosive behavior occur in 2006 (Agriculture), 2013-2014 (GHG), 2002-2010 (Forest), 2001 (Income), and 2006 (Land-use) whereas no episodes of explosive behavior occur for urban population, since the estimated test is insignificant.

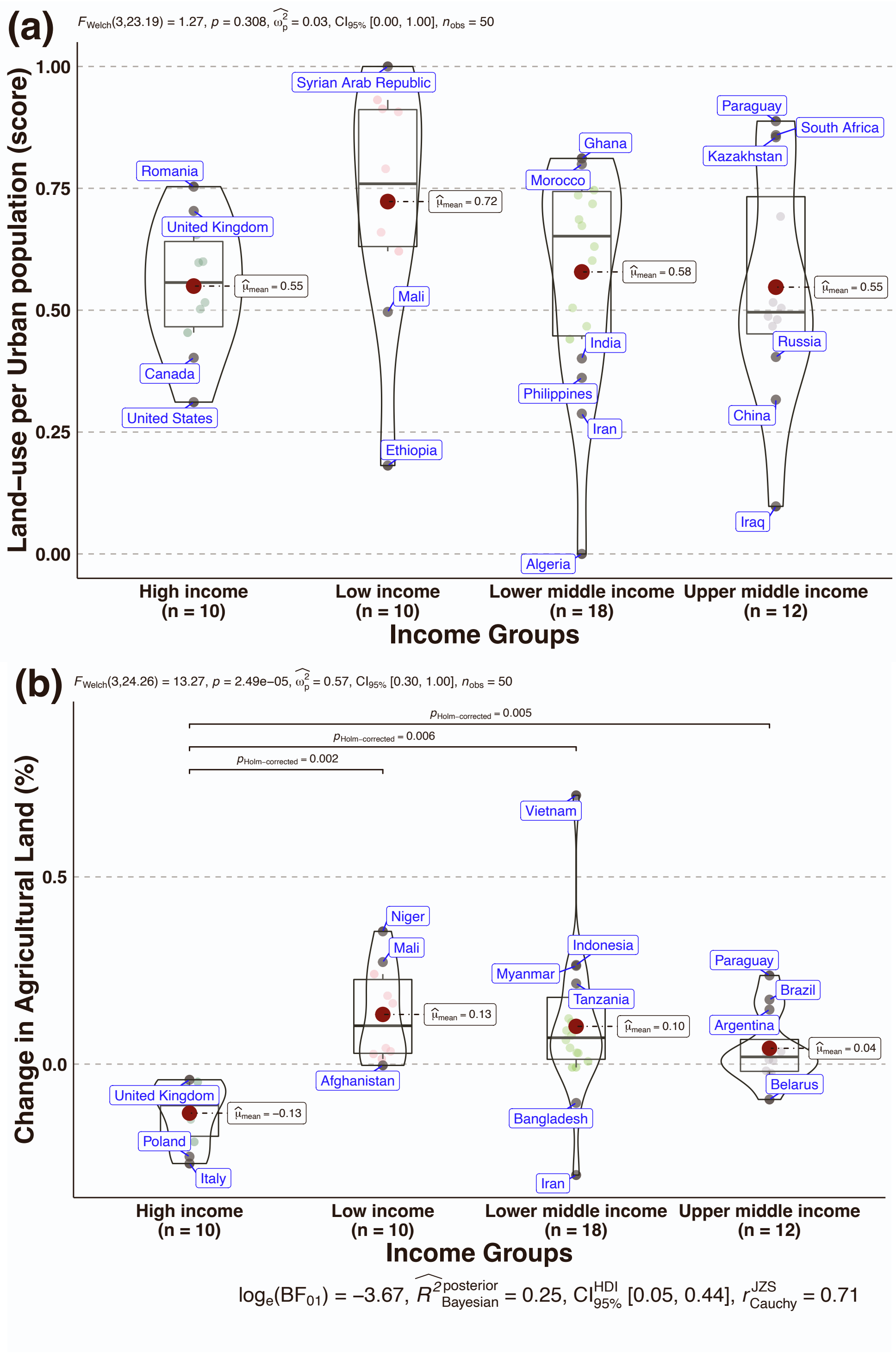

Figure S5. Distribution across income groups (a) Land-use intensity per urban population (b) Change in Agricultural land. Related to Figure 5.

The pairwise test using the Games-Howell technique shows only statistically significant comparisons. (•) represents the within mean across income groups. The output of the frequentist analysis $F_{welch}(.)$ = #, p = #, $\widehat{\omega_p^2}$ = #, $CI_{95\%}$ [#, #], n_{obs} = #, denote the parameter test statistic, significance of the p-value, estimate of the effect size, confidence interval, and number of observations. The output of the Bayesian analysis $log_e(.)$ = #, $\widehat{R^2}^{posterior}_{Bayesian}$ = #, $CI^{HDI}_{95\%}$ [#, #], r^{JZS}_{Cauchy} = #, represents the logarithm of Bayes Factor to test evidence in favor of the null hypothesis over the alternative, R^2 estimate of posterior Bayesian, and prior value.

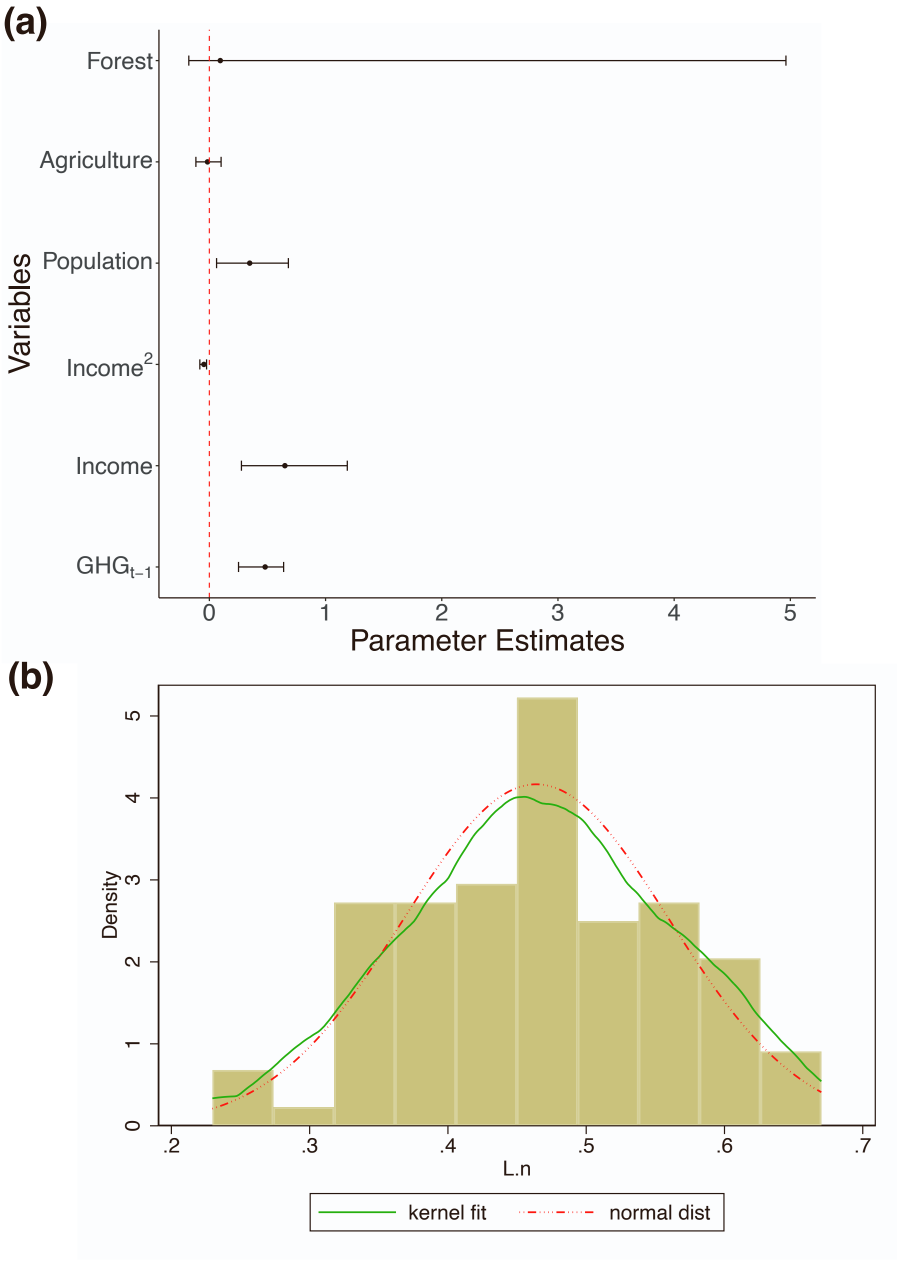

Figure S6. Parameter estimation (a) GHG emissions, income, agriculture, and forest nexus (b) Model validation using bootstrap distribution for all autoregressive coefficients. Related to Figure 6.

The parameter estimates of all variables excluding agricultural and forest land use are statistically significant at *P-value*<0.05. Bootstrap corrected dynamic FE regression (n = 1300) based on Cross-section dependence resampling and analytical heterogeneous initialization to achieve convergence. The estimated model has bootstrapped standard errors, bootstrap 95% (percentile-based) confidence intervals, and statistical inferences performed with non-parametric bootstrap. Residual diagnostics: CD-test (-0.21) & *p-value* (0.836); Pesaran's CADF test (-1.382) & *p-value* (0.997).

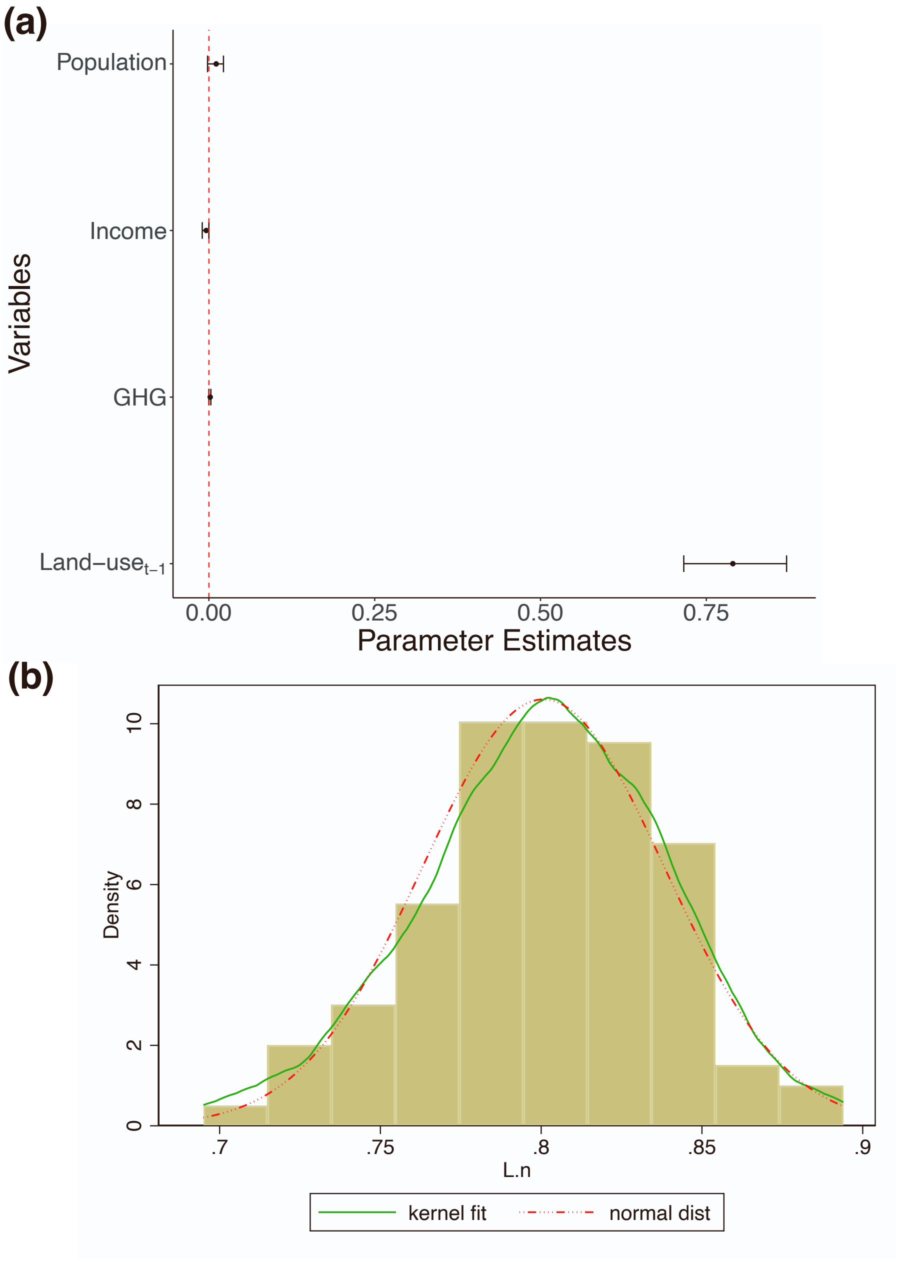

Figure S7. Parameter estimation (a) Land use, GHG emissions, income, and urbanization (b) Model validation using bootstrap distribution for all autoregressive coefficients. Related to Figure 6.

The parameter estimates of all variables excluding income level are statistically significant at *P-value*<0.05. Heterogeneous slope testing—Standard delta test $\tilde{\Delta}$ (23.728, *p<0.01*), adjusted delta test $\tilde{\Delta}_{adj}$ (26.286, *p<0.01*), and HAC robust delta test $\tilde{\Delta}_{HAC}$ (-3.209, *p<0.01*). Bootstrap corrected dynamic FE regression (n = 1300) based on cross-section dependence resampling and analytical heterogeneous initialization to achieve convergence. The estimated model has bootstrapped standard errors, bootstrap 95% (percentile-based) confidence intervals, and statistical inferences performed with non-parametric bootstrap. Residual diagnostics: CD-test (7.24); Pesaran's CADF test (-1.079) & *p-value* (1.000).

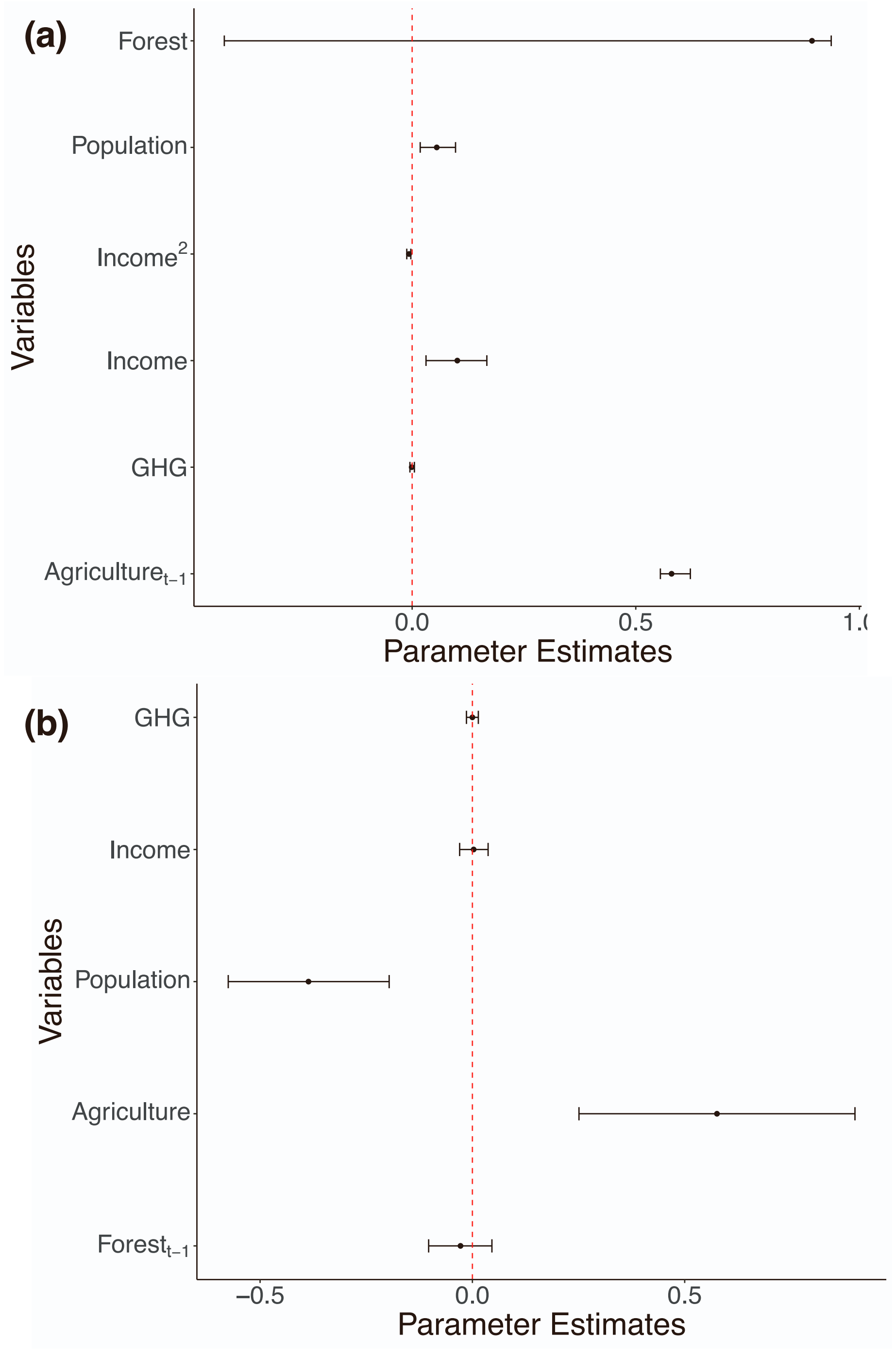

Figure S8. Parameter estimation (a) Agricultural land-EKC hypothesis (b) Nexus among forest, agriculture, urbanization, income, and GHG emissions. Related to Figure 6. The parameter estimates of all variables in (a) excluding GHG emissions are statistically significant at *P-value*<0.05 whereas estimates of all variables in (b) excluding agricultural land use, income, and GHG emissions are statistically significant at *P-value*<0.01. Model validation for (a) was executed using bootstrap distribution for all autoregressive coefficients. Bootstrap corrected dynamic FE regression (n = 1300) based on Cross-section dependence resampling and burn-in initialization to achieve convergence. The estimated model has bootstrapped standard errors, bootstrap 95% (percentile-based) confidence intervals, and statistical inferences performed with non-parametric bootstrap. Residual

diagnostics: CD-test (7.67) & *p-value* (0.000); Pesaran's CADF test (-1.305) & *p-value* (0.999). In contrast, (b) entails heterogeneous slope testing—Standard delta test $\tilde{\Delta}$ (35.882, $p<0.01$), adjusted delta test $\tilde{\Delta}_{adj}$ (40.687, $p<0.01$), and HAC robust delta test $\tilde{\Delta}_{HAC}$ (-6.775, $p<0.01$). Test of endogeneity using robust regression: F(1,49) = 3.690 [verdict: The test for endogeneity confirms the validity of adopting instrumental-variables estimator for the forest model], & $p<0.1$. Residual diagnostics: CD-test (27.52); Pesaran's CADF test (-0.607), & *p-value* (1.000).

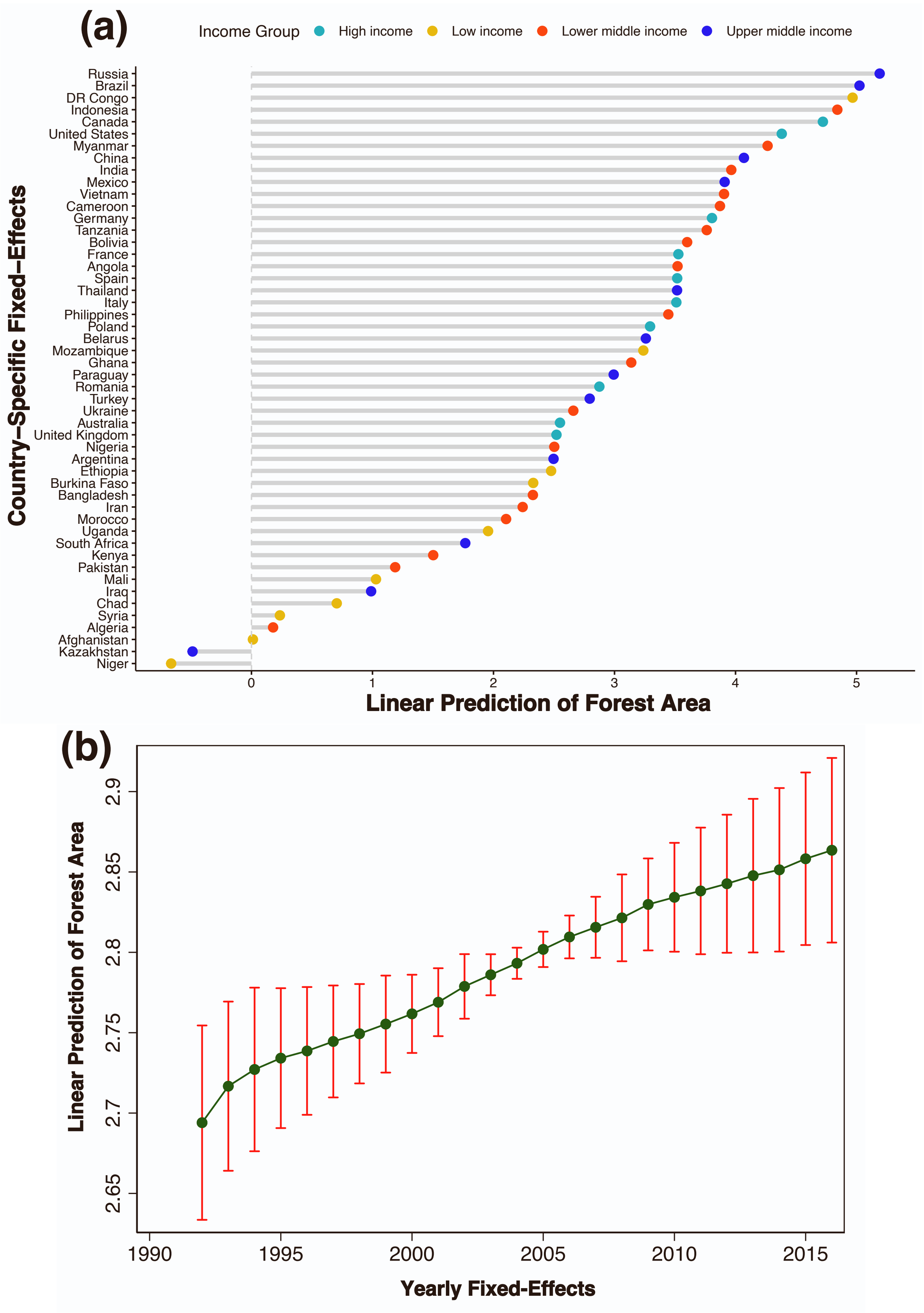

Figure S9. Predictive margins of (a) Country-Specific-Fixed Effects on Forest Area (b) Yearly-Fixed Effects on Forest Area. Related to Figure 6.

The red vertical-bars represent 95% confidence intervals whereas the green dots are the linear predictions.
